# Rural–urban difference in the use of annual physical examination among seniors in Shandong, China: a cross-sectional study

**DOI:** 10.1186/s12939-017-0585-z

**Published:** 2017-05-23

**Authors:** Dandan Ge, Jie Chu, Chengchao Zhou, Yangyang Qian, Li Zhang, Long Sun

**Affiliations:** 1 School of Public Health, Shandong University, Jinan, 250012 China; 2 Shandong Centre for Disease Control and Prevention, Jinan, 250014 China; 3 Collaborative Innovation Center of Social Risks Governance in Health, 44 Wen-hua-xi Road, Jinan, Shandong 250012 China

**Keywords:** Elderly, Annual physical examination, Health service use, Rural–urban difference, China

## Abstract

**Background:**

Regular physical examination contributes to early detection and timely treatment, which is helpful in promoting healthy behaviors and preventing diseases. The objective of this study is to compare the annual physical examination (APE) use between rural and urban elderly in China.

**Methods:**

A total of 3,922 participants (60+) were randomly selected from three urban districts and three rural counties in Shandong Province, China, and were interviewed using a standardized questionnaire. We performed unadjusted and adjusted logistic regression models to examine the difference in the utilization of APE between rural and urban elderly. Two adjusted logistic regression models were employed to identify the factors associated with APE use in rural and urban seniors respectively.

**Results:**

The utilization rates of APE in rural and urban elderly are 37.4% and 76.2% respectively. Factors including education level, exercise, watching TV, and number of non-communicable chronic conditions, are associated with APE use both in rural and urban elderly. Hospitalization, self-reported economic status, and health insurance are found to be significant (*p* < 0.05) predictors for APE use in rural elderly. Elderly covered by Urban Resident Basic Medical Insurance (URBMI) (*p* < 0.05, OR = 1.874) are more likely to use APE in urban areas.

**Conclusions:**

There is a big difference in APE utilization between rural and urban elderly. Interventions targeting identified at-risk subgroups, especially for those rural elderly, are essential to reduce such a gap. To improve health literacy might be helpful to increase the utilization rate of APE among the elderly.

## Background

Population aging has become a worldwide phenomenon, with China among some of the fastest-aging societies as the number of its seniors and life expectancy have been increasing over the past decades. National Bureau of Statistic of China reported that the proportion of China’s population aged 60 and above was 16.2% (221.8 million) at the end of 2015, representing an increase of 5.8% compared with that in 2000 [1, 2]. This demographic change has posed several challenges (i.e., social insurance, and public services) to the individuals, the family, and also the society [3]. Among which, healthcare service is an important issue which deserves concern.

The population aging is accompanied by an increase in prevalence of some diseases, especially of non-communicable chronic diseases (NCDs), such as cerebrovascular disease, heart disease, respiratory disease, diabetes, and hypertension, which place a heavy burden on the seniors and also their families [4]. Furthermore, the increase in the proportion of the older people in a country is found to be a driver of national health expenditure. Previous studies have demonstrated that periodic physical examination is an important and effective measure for illness prevention and health promotion through early detection and timely treatment [5–7]. Moreover, periodic physical examination (i.e., annual physical examination) is found to have a negative correlation with health expenditure among the seniors [8].

Many previous studies have explored the factors associated with the utilization of physical examination in China and found that the physical examination use is influenced by demographic characteristics, such as gender, education, income level, and marital status [9–11]. Ye and Wang found that the use of physical examination was higher among those elderly with poorer self-rated health in rural Wenling, China [12]. A study by Li et al. found that health literacy was an important risk factor for the use of physical examination among urban seniors in Chengdu [13]. However, most of the existing studies only focused on the rural or urban residents. To date, no studies have explored the difference in the use of periodic physical examination between rural and urban residents in China.

To remedy this situation, the present study aims to explore the difference in the use of annual physical examination (APE) between rural and urban areas in China. To do so, we have following specific objectives. First, we will compare the difference in use of APE between rural and urban seniors in China. Second, we will identify the risk factors associated with the use of APE in rural and urban elderly respectively.

## Methods

### Subjects

This cross-sectional study was conducted in Shandong province from November, 2011 to January, 2012. Shandong has the second largest total population in China, and its older people (60+) accounted for 15% of the total population (about 970million) in 2012 [14]. We employed a 3-stage cluster sampling method to selected participants. First, all of the districts and counties in Shandong were divided into three groups according to the GDP per capita in 2011. Second, one district and one county were randomly selected from each group. Three districts (Huaiyin, Dongchangfu, Zhangdian) and three counties (Qufu, Chiping, Leling) were chosen as study sites. Likewise, three sub-districts and three townships were then selected from each sampling district or county according the GDP per capita, respectively. Third, three communities or three villages were randomly selected from each selected sub-district and township (27 communities, and 27 villages). All of the elderly households of the sampling communities or villages were recruited into our survey. In total, 3922 participants were included in this study.

### Variables

#### Social demographic characteristics

Social demographic characteristics included gender, age, education, marital status, self-reported economic status, health insurance, and the distance to the nearest health providers. The participants’ age was divided into three groups: 60−, 70−, and, 80+ years. Other demographic characteristics were categorized as follows: gender (male vs. female), education level (illiteracy or semiliterate, primary school, junior school, and senior school or above), residence (rural vs. urban), marital status (single vs. couple), economic status (poor, normal, and good), health insurance (Urban Employee Basic Medical Insurance, UEBMI; Urban Resident Basic Medical Insurance, URBMI; New cooperative medical scheme, NCMS; others; and none), and the distance to the nearest health providers (<1 km, 1 km+).

#### Health behaviors

We collected health behavior information of the participants, including smoking, drinking, exercise, watching TV. These characteristics were categorized as follows: smoking (yes vs. no), drinking (at least 1 time a week vs. no or less than 1 time a week), exercise (at least 3 times a week vs. less than 3 times a week), watching TV (yes vs. no).

#### Physical health

We collected the information about physical health, including number of NCDs (none, 1, 2, and, 3 or above), and hospitalization (yes vs. no).

#### ADL

This study applied Activity of Daily Living Scale (ADL) which consists of 14 questions to evaluate the ability to perform activities of daily living. Total score is 14 to 56, and the higher total score indicates worse ADL status [15]. A total score of 14 was suggestive of normal, which was coded into 1; a total score of 15 ~ 21 was suggestive of mild disabilities, which was coded into 2; and a total score of 22 and above was suggestive of apparent obstacles, which was coded into 3. The Cronbach’s α of the ADL in this study was 0.95.

#### APE

APE was measured by a question of “Did you have any physical examinations in the past twelve months”. The answer included “yes” [1] and “no” (0).

### Statistical analysis

All statistical analyses were performed using SPSS 16.0. We employed unadjusted model to identify potential factors associated with APE use among the elderly. Two models, one unadjusted and one adjusted regression model, were used to examine the rural–urban difference in the APE use. Further, two binary logistic regression models were used to identify the factors associated with APE use in rural and urban elderly respectively.

## Results

Of all respondents, rural elderly account for 54.9% (2153), urban elderly account for 45.1% (1769). And 37.4% of rural elderly use APE, 76.2% of urban elderly use APE (Table 1). Results from the unadjusted model are presented in the Table 1. Those who live in urban areas (*p* < 0.05), who have higher educational level (*p* < 0.05), who have a good economic status (*p* < 0.05), who often do exercise (*p* < 0.05), who watch TV (*p* < 0.05), who have one or more types of NCDs (*p* < 0.05), who have inpatient services (*p* < 0.05),who are covered by UEBMI (*p* < 0.005) or URBMI (*p* < 0.05) are more likely to use APE.

**Table 1:** Factors associated with annual physical examination among the seniors in Shandong, China, 2012

Characteristics	Total (N/%)	APE^a^ Use (N/%)		Unadjusted model			Adjusted model		
		Yes	No	OR	95%CI	*P*	OR	95%CI	*P*
Observations	3922	2154 (54.9)	1768 (45.1)						
Region									
Rural	2153 (54.9)	806 (37.4)	1347 (62.6)	1.00			1.00		
Urban	1769 (45.1)	1348 (76.2)	421 (23.8)	5.35	4.65–6.15	*0.000*	2.76	2.26–3.36	*0.000*
Gender							NA^b^		
Male	1822 (46.5)	1018 (55.9)	804 (44.1)	1.00					
Female	2100 (53.5)	1136 (54.1)	964 (45.9)	0.93	0.82–1.05	0.265			
Age						0.082	NA		
60−	2569 (65.5)	1392 (54.2)	1177 (45.8)	1.00					
70−	1121 (28.6)	644 (57.4)	477 (42.6)	1.14	0.99–1.31	0.067			
80+	232 (5.9)	118 (50.9)	114 (49.1)	0.87	0.66–1.14	0.331			
Education						*0.000*			*0.000*
Illiteracy or semiliterate	1736 (44.3)	758 (43.7)	978 (56.3)	1.00			1.00		
Primary school	1175 (30.0)	680 (57.9)	495 (42.1)	1.77	1.52–2.05	*0.000*	1.30	1.10–1.55	*0.002*
Junior school	597 (15.2)	391 (65.5)	206 (34.5)	2.44	2.01–2.97	*0.000*	1.30	1.04–1.63	*0.020*
Senior school or above	414 (10.6)	325 (78.5)	89 (21.5)	4.71	3.65–6.06	*0.000*	1.95	1.45–2.62	*0.000*
Marital status									
Single	817 (20.8)	409 (50.1)	408 (49.9)	1.00			1.00		
Couple	3105 (79.2)	1745 (56.2)	1360 (43.8)	1.28	1.09–1.49	*0.002*	1.01	0.84–1.20	0.936
Economic status						*0.000*			0.060
Poor	677 (17.3)	298 (44.0)	379 (56.0)	1.00			1.00		
Normal	2319 (59.1)	1251 (53.9)	1068 (46.1)	1.49	1.25–1.77	*0.000*	1.21	1.00–1.47	0.046
Good	926 (23.6)	605 (65.3)	321 (34.7)	2.39	1.95–2.93	*0.000*	1.31	1.03–1.65	*0.023*
Smoking									
No	3041 (77.5)	1741 (57.3)	1300 (42.7)	1.00			1.00		
Yes	881 (22.5)	413 (46.9)	468 (53.1)	0.65	0.56–0.76	*0.000*	0.89	0.74–1.07	0.221
Drinking (time/a week)									
<1	3243 (82.7)	1805 (55.7)	1438 (44.3)	1.00			1.00		
≥1	679 (17.3)	349 (51.4)	330 (48.6)	0.84	0.71–0.99	*0.043*	0.93	0.76–1.14	0.505
Exercise (time/a week)									
<3	1514 (38.6)	633 (41.8)	881 (58.2)	1.00			1.00		
≥3	2408 (61.4)	1521 (63.2)	887 (36.8)	2.38	2.09–2.72	*0.000*	1.53	1.32–1.78	*0.000*
Watching TV									
No	470 (12.0)	179 (38.1)	291 (61.9)	1.00			1.00		
Yes	3452 (88.0)	1975 (57.2)	1477 (42.8)	2.17	1.78–2.65	*0.000*	1.69	1.34–2.12	*0.000*
Number of NCDs^c^						*0.000*			*0.001*
None	1338 (34.1)	649 (48.5)	689 (51.5)	1.00			1.00		*0.001*
1	1781 (45.4)	1006 (56.5)	775 (43.5)	1.37	1.19–1.58	*0.000*	1.29	1.10–1.52	*0.002*
2	582 (14.8)	354 (60.8)	228 (39.2)	1.64	1.35–2.00	*0.000*	1.41	1.12–1.77	*0.003*
3+	221 (5.6)	145 (65.6)	76 (34.4)	2.02	1.50–2.72	*0.000*	1.78	1.26–2.52	*0.001*
Hospitalization									
No	3355 (85.5)	1790 (53.4)	1565 (46.6)	1.00			1.00		
Yes	567 (14.5)	364 (64.2)	203 (35.8)	1.56	1.30–1.88	*0.000*	1.19	0.96–1.48	0.101
ADL^d^						*0.000*			0.247
1	2854 (72.8)	1638 (57.4)	1216 (42.6)	1.00			1.00		
2	630 (16.1)	331 (52.5)	299 (47.5)	0.82	0.69–0.97	*0.026*	1.03	0.85–1.26	0.716
3	438 (11.2)	185 (42.2)	253 (57.8)	0.54	0.44–0.66	*0.000*	0.83	0.65–1.05	0.133
The distance to the nearest health institutions (km)									
<1	3639 (92.8)	2024 (55.6)	1615 (44.4)	1.00			1.00		
≥1	283 (7.2)	130 (45.9)	153 (54.1)	0.67	0.53–0.86	*0.002*	0.98	0.75–1.28	0.893
Type of health insurance^e^						*0.000*			*0.000*
None	113 (2.9)	57 (50.4)	56 (49.6)	1.00			1.00		
UEBMI	808 (20.6)	658 (81.4)	150 (18.6)	4.31	2.86–6.48	*0.000*	2.13	1.36–3.33	*0.001*
URBMI	470 (12.0)	370 (78.7)	100 (21.3)	3.63 `	2.36–5.58	*0.000*	2.18	1.37–3.46	*0.001*
NCMS	2507 (63.9)	1053 (42.0)	1454 (58.0)	0.71	0.48–1.03	0.077	1.15	0.75–1.74	0.511
Others	24 (0.6)	16 (66.7)	8 (33.3)	1.96	0.77–4.95	0.153	1.68	0.61–4.64	0.311

Note: The italics indicate significance

^a^ *APE* annual physical examination

^b^ *NA* not applicable

^c^ *NCDs* Non-communicable chronic diseases

^d^ *ADL* activity of daily living

^e^ *UEBMI*, Urban Employee Basic Medical Insurance, *URBMI* Urban Resident Basic Medical Insurance, *NCMS* New cooperative medical scheme

The adjusted model shows that when controlling for other variables, the utilization rate of APE in the urban elderly is still statistically higher than that in rural elderly (AOR = 2.761, *p* = 0.000). In addition, the results also show that those who have higher educational level (*p* < 0.05), who often do exercise (*p* = 0.000), who watch TV (*p* = 0.000), who have one or more types of NCDs (*p* < 0.005), who are covered by UEBMI (*p* = 0.001) or URBMI (*p* = 0.001) are more likely to use APE.

Two adjusted binary logistic regression models are conducted to identify the factors associated with the use of APE in rural (Table 2) and urban seniors (Table 3) respectively. Four factors are found to be statistically associated with the APE use (*p* < 0.05) both in rural and urban elderly, including education level, exercise, watching TV, and the number of NCDs. Among rural elderly, those who experienced hospitalization use (AOR = 1.438, *p* = 0.011), who report good economic status (AOR = 1.600, *p* = 0.002), who are covered by some types of insurance (not NCMS) (AOR = 3.419, *p* = 0.010) were more likely to use APE. Among urban elderly, those who are covered URBMI (AOR = 1.874, *p* = 0.025) are more likely to use APE.

**Table 2:** Factors associated with the use of physical examination among rural elderly in Shandong, China (2012)

Characteristics	Total (N/%)	APE^a^ Use (N/%)		Unadjusted model			Adjusted model		
		Yes	No	OR	95%CI	*P*	OR	95%CI	*P*
Observations	2153	806 (37.4)	1347 (62.6)						
Gender							NA^b^		
Male	1045 (48.5)	396 (37.9)	649 (62.7)	1.00					
Female	1108 (51.5)	410 (37.0)	698 (63.0)	0.96	0.80–1.14	0.669			
Age						0.326	NA		
60−	1468 (68.2)	563 (38.4)	905 (61.6)	1.00					
70−	550 (25.5)	199 (36.2)	351 (63.8)	0.91	1.74–1.11	0.371			
80+	135 (6.3)	44 (32.6)	91 (67.4)	0.77	0.53–1.13	0.188			
Education						*0.000*			0.122
Illiteracy or semiliterate	1206 (56.0)	409 (33.9)	797 (66.1)	1.00			1.00		
Primary school	631 (29.3)	252 (39.9)	379 (60.1)	1.29	1.06–1.58	*0.011*	1.13	0.92–1.39	0.238
Junior school	215 (10.0)	90 (54.5)	125 (58.1)	1.40	1.04–1.88	*0.025*	1.16	0.85–1.58	0.342
Senior school or above	101 (4.7)	55 (54.5)	46 (45.5)	2.33	1.54–3.50	*0.000*	1.66	1.06–2.58	*0.025*
Marital status							NA		
Single	504 (23.4)	171 (33.9)	333 (66.1)	1.00					
Couple	1649 (76.6)	635 (38.5)	1014 (61.5)	1.22	0.98–1.50	0.630			
Economic status						*0.000*			*0.010*
Poor	448 (20.8)	135 (30.1)	313 (69.9)	1.00			1.00		
Normal	1338 (62.1)	504 (37.7)	834 (62.3)	1.40	1.11–1.76	*0.004*	1.24	0.97–1.58	0.074
Good	367 (17.0)	167 (45.5)	200 (54.5)	1.93	1.45–2.58	*0.000*	1.60	1.18–2.16	*0.002*
Smoking							NA		
No	640 (70.3)	570 (37.7)	943 (62.3)	1.00					
Yes	1513 (29.7)	236 (36.9)	404 (63.1)	0.96	0.79–1.17	0.726			
Drinking (time/a week)							NA		
<1	1719 (79.8)	649 (37.8)	1070 (62.2)	1.00					
≥1	434 (20.2)	157 (36.2)	277 (63.8)	0.93	0.75–1.16	0.544			
Exercise (time/a week)									
<3	1051 (51.2)	359 (32.6)	743 (67.4)	1.00			1.00		
≥3	1102 (48.8)	447 (42.5)	604 (57.5)	1.53	1.28–1.82	*0.000*	1.41	1.18–1.69	*0.000*
Watching TV									
No	304 (14.1)	75 (24.7)	229 (75.3)	1.00			1.00		
Yes	1849 (85.9)	731 (39.5)	1118 (60.5)	1.99	1.51–2.63	*0.000*	1.72	1.28–2.30	*0.000*
Number of NCDs^c^						*0.001*			*0.000*
None	808 (37.5)	267 (33.1)	541 (67.0)	1.00			1.00		
1	963 (44.7)	368 (38.2)	595 (61.8)	1.25	1.03–1.52	*0.024*	1.32	1.07–1.62	*0.008*
2	278 (12.9)	123 (44.2)	155 (55.8)	1.60	1.21–2.12	*0.001*	1.78	1.32–2.40	*0.000*
3+	104 (4.8)	48 (46.2)	56 (53.8)	1.73	1.15–2.62	*0.009*	2.02	1.30–3.14	*0.002*
Hospitalization									
No	1900 (88.2)	688 (36.2)	1212 (63.8)	1.00			1.00		
Yes	253 (11.8)	688 (46.6)	135 (53.4)	1.54	1.18–2.00	*0.001*	1.43	1.08–1.90	*0.011*
ADL^d^						0.069			0.266
1	1448 (67.3)	561 (38.7)	887 (61.3)	1.00			1.00		
2	399 (18.5)	148 (37.1)	251 (62.9)	0.93	0.74–1.17	0.548	0.92	0.72–1.17	0.502
3	306 (14.2)	97 (31.7)	209 (68.3)	0.73	0.56–0.95	*0.021*	0.78	0.58–1.05	0.109
The distance to the nearest health institutions (km)							NA		
Less than 1 km	1933 (89.8)	727 (37.6)	1206 (62.4)	1.00					
1 km+	220 (10.2)	79 (35.9)	141 (64.1)	0.92	0.69–1.24	0.621			
Type of health insurance^e^						*0.000*			*0.014*
None	38 (1.8)	8 (21.1)	30 (78.9)	1.00			1.00		
NCMS	2036 (94.6)	750 (36.8)	1286 (63.2)	2.18	0.99–4.79	0.051	1.82	0.81–4.07	0.144
Others	79 (3.7)	48 (60.8)	31 (39.2)	5.80	2.35–14.29	*0.000*	3.41	1.34–8.71	*0.010*

Note: Of the 2153 rural participants, 53(2.46%) older people had retired from urban areas

The italics indicate significance

^a^ *APE* annual physical examination

^b^ *NA* not applicable

^c^ *NCDs* Non-communicable chronic diseases

^d^ *ADL* activity of daily living

^e^ *NCMS* New cooperative medical scheme

**Table 3:** Factors associated with the use of physical examination among urban elderly in Shandong, China (2012)

Characteristics	Total (N/%)	APE^a^ Use (N/%)		Unadjusted model			Adjusted model		
		Yes	No	OR	95%CI	*P*	OR	95%CI	*P*
Observations	1769	1348 (76.2)	421 (23.8)						
Gender									
Male	777 (43.9)	622 (80.1)	155 (19.9)	1.00			1.00		
Female	992 (56.1)	726 (53.9)	266 (26.8)	0.68	0.54–0.85	*0.001*	0.83	0.64–1.07	0.166
Age						0.486	NA^b^		
60−	1101 (62.2)	829 (75.3)	272 (24.7)	1.00					
70−	571 (32.2)	445 (77.9)	126 (22.1)	1.15	0.91–1.47	0.230			
80+	97 (5.5)	74 (76.3)	23 (23.7)	1.05	0.64–1.71	0.828			
Education						*0.000*			*0.001*
Illiteracy or semiliterate	530 (30.0)	349 (65.8)	181 (34.2)	1.00			1.00		
Primary school	544 (30.8)	428 (78.7)	116 (21.3)	1.91	1.45–2.51	*0.000*	1.59	1.18–2.13	*0.002*
Junior school	382 (21.6)	301 (78.8)	81 (21.2)	1.92	1.42–2.62	*0.000*	1.44	1.03–2.03	*0.033*
Senior school or above	313 (17.7)	270 (86.3)	43 (13.7)	3.25	2.25–4.70	*0.000*	2.26	1.49–3.44	*0.000*
Marital status							NA		
Single	313 (17.7)	238 (76.0)	75 (24.0)	1.00					
Couple	1456 (82.3)	1110 (76.2)	346 (23.8)	1.01	0.75–1.34	0.941			
Economic status						0.101			0.520
Poor	229 (12.9)	163 (71.2)	66 (28.8)	1.00			1.00		
Normal	981 (55.5)	747 (76.1)	234 (23.9)	1.29	0.93–1.78	0.118	1.19	0.85–1.66	0.305
Good	559 (31.6)	438 (78.4)	121 (21.6)	1.46	1.03–2.07	*0.032*	1.07	0.74–1.56	0.692
Smoking							NA		
No	1528 (86.4)	177 (73.4)	64 (26.6)	1.00					
Yes	241 (13.6)	1171 (76.6)	357 (23.4)	1.18	0.87–1.61	0.280			
Drinking (time/a week)							NA		
<1	1524 (86.2)	192 (78.4)	53 (21.6)	1.00					
≥1	245 (13.8)	1156 (85.8)	368 (24.1)	1.15	0.83–1.59	0.391			
Exercise (time/a week)									
<3	1357 (23.3)	274 (66.5)	138 (33.5)	1.00			1.00		
≥3	412 (76.7)	1074 (79.1)	283 (20.9)	1.91	1.49–2.43	*0.000*	1.83	1.42–2.37	*0.000*
Watching TV									
No	166 (9.4)	104 (62.7)	62 (37.3)	1.00			1.00		
Yes	1603 (90.6)	1244 (77.6)	359 (22.4)	2.06	1.47–2.89	*0.000*	1.59	1.11–2.28	*0.011*
Number of NCDs^c^						*0.026*			0.074
None	530 (30.0)	382 (72.1)	148 (27.9)	1.00			1.00		
1	818 (46.2)	638 (78.0)	180 (22.0)	1.37	1.06–1.76	*0.014*	1.32	1.01–1.73	*0.037*
2	304 (17.2)	231 (76.0)	73 (24.0)	1.22	0.88–1.69	0.218	1.08	0.76–1.53	0.651
3+	117 (6.6)	97 (82.9)	20 (17.1)	1.87	1.12–3.15	*0.017*	1.74	1.00–3.02	*0.048*
Hospitalization							NA		
No	1455 (82.2)	1102 (75.7)	353 (24.3)	1.00					
Yes	314 (17.8)	246 (78.3)	68 (21.7)	1.15	0.86–1.55	0.326			
ADL^d^						*0.021*			0.218
1	1406 (79.5)	1077 (76.6)	329 (23.4)	1.00			1.00		
2	231 (13.1)	183 (79.2)	48 (20.8)	1.16	0.82–1.63	0.381	1.34	0.93–1.94	0.108
3	132 (7.5)	88 (66.7)	44 (33.3)	0.61	0.41–0.89	*0.012*	0.91	0.60–1.39	0.692
The distance to the nearest health institutions (km)						NA			
<1	1706 (96.4)	1297 (76.0)	409 (24.0)	1.00					
≥1	63 (3.6)	51 (81.0)	12 (19.0)	1.34	0.70–2.53	0.369			
Type of health insurance^e^						*0.000*			*0.000*
None	75 (4.2)	49 (65.3)	26 (34.7)	1.00			1.00		
UEBMI	755 (42.7)	622 (82.4)	133 (17.6)	2.48	1.48–4.13	*0.000*	1.70	0.98–2.92	0.056
URBMI	454 (25.7)	362 (79.7)	92 (20.3)	2.08	1.23–3.53	*0.006*	1.87	1.08–3.23	*0.025*
Others	485 (27.4)	315 (64.9)	170 (35.1)	0.98	0.59–1.63	0.948	0.95	0.56–1.61	0.861

Note: The italics indicate significance

^a^ *APE* annual physical examination

^b^ *NA* not applicable

^c^ *NCDs* Non-communicable chronic diseases

^d^ *ADL* activity of daily living

^e^ *UEBMI* Urban Employee Basic Medical Insurance, *URBMI* Urban Resident Basic Medical Insurance

## Discussion

We find that 37.4% of the rural elderly and 76.2% of the urban elderly used APE. The prevalence in APE use among the rural elderly in this study is much lower than that reported among the elderly by Sun et al. (63.5%), which was conducted in rural areas of four provinces or municipalities (Zhejiang, Henan, Qinghai, and Chongqing) in China [16]. It is a little higher than the utilization rate of 10.5% for the rural elderly in Anhui province, China [17]. Likewise, the rate of the APE use among urban seniors of our study is a little lower than that among the elderly in urban Chengdu (84.1%) [13]. But it is higher than the reported rate of 54.1% in urban Xuzhou [18]. In different settings, there is a large variation of APE use both in rural and urban elderly in China.

Since the implementation of the new health care reform scheme of China in 2009, APE has been provided free to the seniors aged 65 and above in rural and urban communities. In some regions, this program is expanded to those aged 60 and above [19, 20]. The program aims to promote good health for the seniors and reduce medical burdens through APE. Some studies found that the proportion of the elderly beneficiaries increased rapidly since the initiation of the program [21, 22]. Unexpectedly, despite the free services, there is still a large proportion of the elderly have not yet used the APE. Among the rural seniors, the utilization rate of the APE is much lower, remaining only at about 37%. This finding indicates an urgent need for studies to identify the barriers for the use of the APE among the elderly in China, especially in rural areas.

The result of this study demonstrates a big difference in APE utilization among the seniors between rural and urban China, which was consistent with the findings from some international studies [23, 24]. A study in Texas showed that rural residents were less likely to receive preventive care than their counterparts in urban areas [25]. There might be three explanations for this finding. One explanation is that APE services are poorly accessible in rural areas. Despite the services are free of charge, the long distance to the designated health providers will probably discourage the seniors’ use of APE, especially for those with NCDs or poor ADL, due to the transportation cost and substantial time incurred [24]. Another explanation is that most of the rural China’s seniors are afraid of being detected some potential serious diseases, and may refuse to use the APE services provided free by the governments. Third, most of the organizations in which the elderly ever worked are still responsible for the arrangement of periodic physical examinations even after their retirement, and will remind them repeatedly when their APE is due [16]. The rural elderly, by contrast, will be seldom reminded to use APE.

Similar with previous studies, the current study shows that educational level, exercise, watching TV, NCDs are factors associated with APE use both in rural and urban elderly. Many studies indicated that those elderly with higher educational level also had higher level of health literacy and ability to access health services [26, 27]. A study about the effect of health literacy on utilization of essential public health services among the elderly indicated to improve the health literacy among the elderly could effectively increase the utilization of essential public health services including physical examination [28]. Some previous studies also indicated that watching TV was the most important way to acquire health knowledge for the Chinese people. Watching TV has been proved to be effective to increase people’s health literacy in China [29–31]. A study by Lai and Kalyniak found that having one or more illness would increase the probability of having APE significantly among those aging Chinese Canadians, which was in agreement with the finding of this study [32]. In China, periodic physical examination is an effective way to monitor and manage NCDs. Thus, people who had NCDs (i.e., hypertension, diabetes) might have a higher utilization rate of APE [33].

Interestingly, the current study indicates that the rural elderly who used inpatients services are found to have higher utilization rate of APE, which is inconsistent with previous studies. A study in Japan indicated an association between an increased use of check-ups and reduced use of hospital inpatient services [34]. Tian et al. also found that people who received preventive care services had lower probabilities of hospitalization use in Taiwan. One explanation for this finding might be that the rural elderly who use APE are more aware and better informed of their health conditions and thus have more frequent visits to hospitals, including inpatient services. Further, economic status of the rural elderly is found to be associated with the APE use, which is in agreement with some previous studies [10, 12, 35].

This study has some limitations. First, the data and relevant analyses showed here derive from a cross-sectional survey, and the relationship between identified factors and APE use cannot be interpreted as cause and effect. Second, the information including economic status and some other variables were self-reported, leading to the possibility of self-report bias. Third, we did not decompose the contribution of different components of explanatory variables in the current study, and will have a more explicit analysis in the follow-up study.

## Conclusion

This study demonstrates a big difference in the use of APE between rural (37.4%) and urban seniors (76.2%) in China. Despite being provided free, there is still a large proportion of the elderly who do not use the APE, which highlights a need to identify the barriers to the APE utilization, especially in rural China. Educational level, number of NCDs, and some other factors are found to be associated with APE use both in rural and urban elderly. Further, hospitalization, self-reported economic status are predictors for APE use in rural elderly. Based on these findings, some recommendations can be given. First, the government should develop targeting health promotion policies to improve health literacy among the elderly. Second, the communities should pay close attention to those identified at-risk subgroups so as to increase the APE use among the elderly.

## Abbreviations

ADL: Activity of daily living
APE: Annual physical examination
NCDs: Non-communicable chronic disease
NCMS: New cooperative medical scheme
UEBMI: Urban employee basic medical insurance
URBMI: Urban resident basic medical insurance.
